# Orthodontic Forced Eruption of Permanent Anterior Teeth with Subgingival Fractures: A Systematic Review

**DOI:** 10.3390/ijerph182312580

**Published:** 2021-11-29

**Authors:** Elisabeth Reichardt, Ralf Krug, Michael M. Bornstein, Jürgen Tomasch, Carlalberta Verna, Gabriel Krastl

**Affiliations:** 1 Department of Pediatric Oral Health and Orthodontics, University Center for Dental Medicine UZB, University of Basel, Mattenstr. 40, 4058 Basel, Switzerland; Carlalberta.Verna@uzb.ch; 2 Department of Conservative Dentistry and Periodontology, University of Würzburg, Pleicherwall 2, 97070 Würzburg, Germany; Krug_R@ukw.de (R.K.); Krastl_G@ukw.de (G.K.); 3 Department of Oral Health & Medicine, University Center for Dental Medicine UZB, University of Basel, Mattenstr. 40, 4058 Basel, Switzerland; Michael.Bornstein@uzb.ch; 4 Department of Molecular Bacteriology, Helmholtz Centre for Infection Research (HZI), IInhoffenstraße 7, 38124 Braunschweig, Germany; Juergen.Tomasch@helmholtz-hzi.de

**Keywords:** orthodontic forced eruption, crown-root fracture, subgingival defects, dental hard tissue defects, dental trauma, root resorption

## Abstract

(1) Background: To assess orthodontic forced eruption (OFE) as a pre-restorative procedure for non-restorable permanent teeth with subgingival dental hard tissue defects after dental trauma. (2) Methods: A systematic electronic search of three databases, namely, MEDLINE, Cochrane Library, and EMBASE, revealed a total of 2757 eligible publications. Randomized controlled clinical trials (RCT), retro- and prospective clinical studies, or case series (with a minimum of three patients) were reviewed. (3) Results: Thirteen full-text papers were included: one RCT, one prospective clinical trial, two retrospective cohort studies, and nine case series. Within case series, statistical significance between age and cause of fracture (*p* < 0.03) was determined. The mean extrusion rate of OFE was 1.5 mm a week within a four to six weeks treatment period followed by retention. Three OFE protocols for maxillary single teeth are available: 1. OFE without migration of gingiva and alveolar bone, 2. OFE with gingival migration and slight alveolar bone migration, and 3. OFE with migration of both gingiva and alveolar bone. (4) Conclusions: The current state of the evidence suggests that OFE is a feasible pre-treatment option for non-restorable permanent teeth. OFE can promote the migration of tooth surrounding hard and soft tissues in the esthetic zone. Root resorption does not seem to be a relevant side effect of OFE.

## 1. Introduction

Restorative treatment of teeth with crown-root fractures (CRF) is challenging due to the subgingival or crestal location of the defects. In maxillary incisors, fracture lines typically extend para- or supragingivally on the buccal side to a subgingival and sometimes subcrestal position on the palatal side.

There are different approaches to the treatment of such teeth, including reattachment of the coronal fragment, direct composite restoration of accessible fractures and surgical crown lengthening (SCL), or extrusion of the root segment [1,2]. In the case of restoring teeth conventionally, the biologic width with approximately 2 mm and a ferrule design with 2 mm are deemed to be ideal distances from the alveolar crest up to the coronal extent of the remaining dental hard tissue. Thus, an adequate so-termed supracrestal tissue attachment (STA) can develop [3,4].

The goal of SCL is to increase the amount of supragingival tooth structure for restorative or esthetic purposes, which is generally accomplished by periodontal flap surgery and osteotomy [5]. One systematic review demonstrated that SCL can predictably increase crown length, but tissue rebound is likely to occur, particularly during the first three postoperative months [6]. Moreover, the esthetic outcome might be compromised by clinical and esthetic alterations, which can affect both adjacent and non-adjacent teeth [4]. Thus, SCL should be carefully considered in patients with a high lip-line and excessive gingival appearance [7]. Orthodontic forced eruption (OFE) might facilitate the re-establishment of biologic width and allow restoration margins to be placed on sound dental hard tissue. Further, it can create or maintain a regular gingival contour, thus improving esthetics. Both orthodontic and surgical extrusion can be considered for the treatment of root fragments.

In contrast to the OFE, the so-termed surgical extrusion is a more invasive one-step extrusion using an axial-pulling tool and not affecting tissue migration. Recently, a narrative review highlighted the low level of evidence regarding the outcome of surgically extruded teeth. Nevertheless, there were favorable success rates in all included 11 case series [8]. One systematic review focusing on adverse events of surgical extrusion reported that non-progressive root resorption was the most common with an event rate of 30%, followed by tooth loss (5%), slight mobility (4.6%), marginal bone loss (3.7%), and progressive root resorption (3.3%) [9]. Further, the findings of an experimental study suggested that an axial extrusion technique that avoids compression of the periodontal tissues during extraction might reduce the occurrence of biological complications, particularly root resorptions [10]. OFE is considered to be a more conservative and physiologic technique than surgical extrusion.

OFE was first described by Heithersay as coronal orthodontic movement achieved by applying continuous orthodontic force for the purpose of preserving soft tissues and gaining vertical bone height [11,12]. A sufficient crown-root-ratio (ideally less than 1:1), healthy gingiva, and osseous structures without any pathological signs are required for successful extrusion and favorable esthetic outcomes in crown-root fractured teeth [11]. OFE with a sectional orthodontic appliance is a feasible alternative to surgical extrusion. However, vertical migration of gingiva and bone around the traumatized tooth towards the extrusion force is an inevitable consequence of orthodontic extrusion [7,13]. The extent of migration of marginal gingiva and alveolar bone depends on how rapidly the root is extruded and how much force is used [12,14]. In cases with physiological and esthetically acceptable gingiva levels before the traumatic event, there is usually no need for coronal migration of the tissues. This is considered as an adverse effect, which can be prevented by periodic fiberotomy [15,16]. Besides the conventional technique using arch wires and elastics, there are only a few case reports of magnetic controlled tooth extrusion. Neodymium containing magnets seem to be suitable with respect to its small size and the high magnetic forces [17,18].

A systematic review published five years ago concluded that OFE is an effective approach to the treatment of teeth with crown-root fractures that provides stable results [19]. However, that review was based on the outcome of only three case reports. Hence, the present systematic review was initiated to collect data on this subject from all available relevant literature published up to August 2020. The aim of this review was to assess if OFE can be used as a successful and effective pre-restorative therapy of non-restorable permanent teeth with subgingival dental hard tissue defects after trauma.

## 2. Materials and Methods

### 2.1. Study Design

The research question (focus) was formulated according to the Patient, Intervention, Comparison, Outcomes (PICO) model [20]. The study characteristics and design are summarized in Table 1.

### 2.2. Search Strategy

Three electronic databases (MEDLINE accessed via PubMed, Cochrane Library, and Embase) were used to perform a systematic search for relevant articles. Specific search terms were formulated for each database (Table 2), and the identified articles were stored using dedicated EndNote X9 software (Thomson Reuters; Toronto, ON, Canada). The search was performed for all articles from any time until the date of the search (6 August 2020). All references cited in the identified articles were checked to identify other potentially relevant articles. Finally, the reviewers searched for additional publications by reference tracking the authors identified in the search.

### 2.3. Screening Process

MEDLINE/PubMed, Cochrane Library, and Embase articles were screened using the MeSH terms listed in Table 2. An asterisk was added to the truncated free-text terms to increase the sensitivity of screening (wildcard search).

### 2.4. Eligibility Criteria

During first stage of study selection, titles and abstracts of the articles were screened and evaluated according to the following inclusion criteria:

- Clinical studies or case series;
- Minimum sample size of ≥3 patients;
- Articles written in English or German language.

The exclusion criteria were as follows:

- Case reports <3 patients, systematic reviews, in vitro studies;
- Missing details on the performed treatment (e.g., extrusion force, amount of movement);
- Duplicate article describing the same sample;
- Forced eruption for extraction/implant site development.

### 2.5. Resources Selection

**Figure 1:**
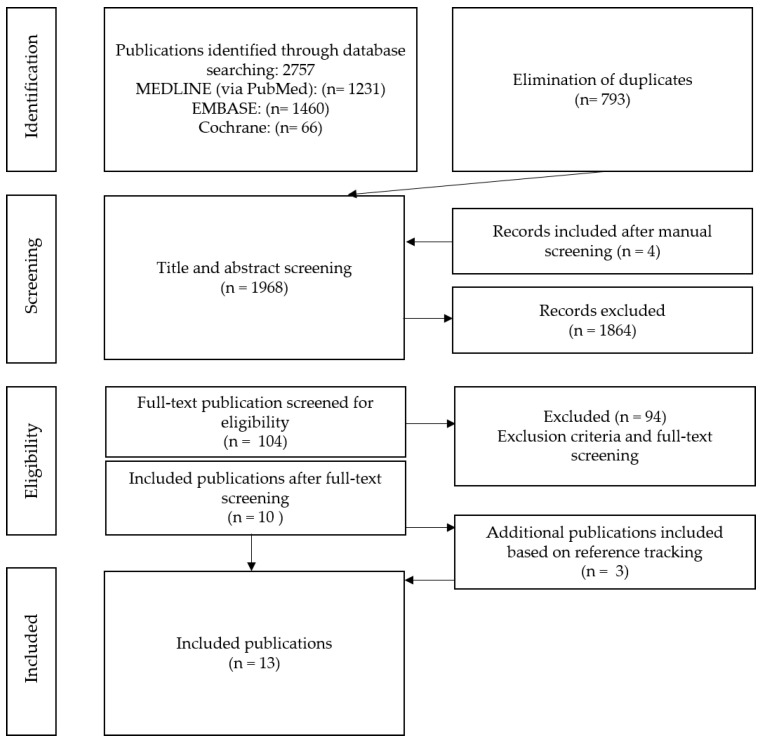
PRISMA flowchart illustrating the study selection process.

The three electronic databases were searched on 6 August 2020. Two reviewers (E.R. and R.K.) independently screened the articles on the basis of the inclusion criteria and also performed a quality assessment of the identified studies (Table 3). After the detection and elimination of duplicates, all remaining articles were screened by title and abstract. If they provided insufficient information for inclusion or exclusion, the full texts were obtained and analyzed. All discrepancies between the two reviewers were discussed with a third author (G.K.) and resolved by consensus (Figure 1).

### 2.6. Data Extraction

Data from the selected articles were extracted using a data extraction form. The reviewers extracted all relevant information, including study design, number of patients, and type of fracture, as well as the orthodontic forced eruption techniques (e.g., types of orthodontic appliances, extrusion forces) and additional procedures (e.g., fiberotomy, periodontal surgery). Furthermore, pulp vitality, root resorption, soft and hard tissue changes, and the amount of tooth movement after OFE of teeth with subgingival fractures were evaluated. The characteristics of the included studies and their outcomes after OFE are summarized in Table 4.

### 2.7. Quality Assessment

A quality assessment was performed applying the instructions recommended in the Cochrane Collaboration Tool for the RCT; the ROBINS-1 scale for the selected clinical trials; the Newcastle Ottawa Scale for the controlled cohort study; and the proposed Pierson, Bradford Hills, and Newcastle Ottawa scale modification-based tool proposed by Murad and colleagues for the nine case series (Table 3) [21,22,23,24,25,26]. The articles included in this systematic review were assessed for several types of bias: selection bias, ascertainment bias, causality bias, reporting bias, allocation bias, blinding of the outcome assessors, incomplete data, and selective notifications of results. The risk of bias (ROB) of each category was graded as low (one point (+)), high (zero points (−)), or unclear (one-half point (+/−)). The quality of all selected clinical trials (ROBINS-1) was graded as low, moderate, or serious ROB [22]. Moreover, an overall ROB of ≥5 points was defined as a lower risk of bias and those with overall ROB of less than five points as a higher risk of bias (Table 3).

### 2.8. Statistical Analysis

Case series data were used to perform two statistical tests. Numerical and categorical variables (e.g., trauma, fracture type, gingival or alveolar bone migration, root resorption) within the case series were assessed by Kruskal–Wallis test concerning the following research questions:

- Does increased force during orthodontic extrusion lead to faster eruption without causing changes in soft and hard tissue as compared to light force?
- Does OFE influence root resorption on the tooth to be extruded?

The chi-squared test of association was performed to determine if there was association between categorical variables (e.g., age, sex, extrusion distance, bracket position, and type of post) within the case series concerning the following research questions:

- Do younger patients (under 50 years) benefit from OFE more than elderly patients (≥50 years) concerning pulp vitality and root resorption?
- Does the use of fixed orthodontic appliances accelerate the extrusion rate of OFE compared to OFE with posts and elastics?
- Does fiberotomy reduce the need for subsequent periodontal surgery after OFE?

Statistical analysis was performed using the Tidyverse 1.3.0. package within the R statistical programming environment, version 3.5.3 (The R Foundation; Vienna, Austria). The level of significance was set at *p* < 0.05.

## 3. Results

### 3.1. Screening Process

The electronic search of the three databases (MEDLINE/PubMed, Cochrane Library, and EMBASE) initially yielded a total of 2757 publications, which were assessed for possible eligibility. After the detection and elimination of duplicates (793), all 1968 remaining articles were screened by title and abstract. Finally, the remaining 13 were included in this systematic review (Figure 1).

### 3.2. Studies Election

#### 3.2.1. Included Studies

The 13 full-text papers included in this review were published between 1972 and 2017 (Table 4). Nine were case series describing OFE of single teeth with crown-root fractures in the esthetic zone. One was a retrospective non-controlled cohort study examining the results of OFE using the Thread Mate System (Coltène-Whaledent Inc.; Mahwah, NJ, USA), featuring a self-threading pin and anchorage wire with a preset extrusion distance [27]. One was a retrospective controlled cohort study of the influence of OFE on pulp status in orthodontic and/or trauma groups [28]. One was a prospective clinical trial evaluating the effectiveness of Nd:YAG laser fiberotomy in crown lengthening by forced eruption [15]. One was an RCT of OFE with or without fiberotomy and root planing [16]. The results were mainly extracted from the case series and showed that forced extrusion can be accomplished by different methods. The case series differed primarily in the number of patients studied. The highest quality work was presented by Malmgren et al. with 32 patients examined (Table 4) [29].

OFE techniques can be divided into three main subgroups depending on the clinical aim and the amount of extrusion force required (weak: 0.2–0.3 N, moderate: >0.3 N and <0.6 N, or strong: >0.6 N).

**Figure 2:**
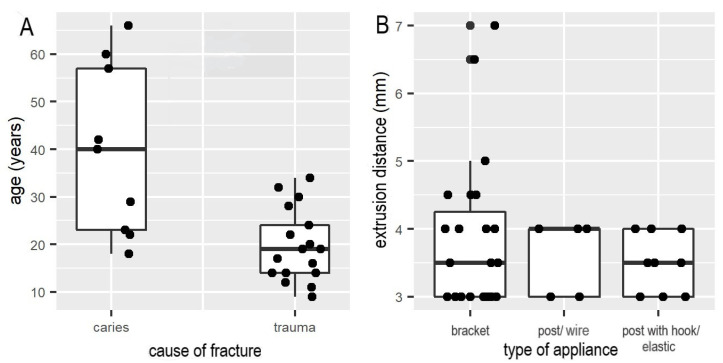
(**A**) Statistical significance of association between age (years) and cause of fracture (*p* < 0.03) within case series. (**B**) Relationship between extrusion distance (mm) and type of appliance (bracket vs. intracanal post with elastic or wire, respectively) within case series.

1. OFE without migration of the gingiva and alveolar bone: OFE without tissue movement requires the use of strong extrusive force (>0.6 N), which was presented in the case series (32 patients) by Malmgren [29]. Another option to prevent gingival and alveolar bone migration is the use of fiberotomy. Both the prospective clinical trial and the RCT showed that coronal movement of the gingival margin can be prevented by sectioning of supracrestal gingival fibers at the beginning and during orthodontic extrusion treatment and simultaneous root planing from the top of the alveolar bone crest [15,16].
2. OFE with gingival migration and slight alveolar bone migration: In the nine case series, this clinical purpose was accomplished by applying weak (0.2–0.3 N) to moderate (>0.3 N and <0.6 N) orthodontic force using round nickel–titanium wires; the mean extrusion distance was 1.5 mm per week. Furthermore, there was no substantial difference in extrusion distance (mm) per week achieved with straight wire appliances, or post with elastics or wires within case series (Figure 2B). However, a trend towards less extrusion distance with the use of elastics was observed (Figure 2B). Analysis of the nine case series showed significant correlations due to shorter extrusion times (up to six weeks) in younger patients (11 to 24 years) versus longer extrusion times per week in adults (up to ten weeks) (*p* < 0.05). Furthermore, there was a significant correlation between caries as a fracture reason and the range of patient’s age from 23 up to 57 years (Figure 2A, *p* < 0.03).
3. OFE with migration of the gingiva and alveolar bone: The third technique, also known as orthodontic implant site development (OISD), is characterized by slow extrusion of the fractured tooth with weak (0.2–0.3 N) to moderate (>0.3 N and <0.6 N) force to induce coronal migration of gingival margin and alveolar bone [13,30]. Simon et al. reported two cases treated with this technique [31].

#### 3.2.2. Methodological Quality

The methodological quality of the studies was mixed. The risk of bias was judged to be high in almost all studies except for the RCT, whose overall risk of bias score was five points using the Cochrane Collaboration Tool [21]. However, the study by Carvalho et al. did not meet all the criteria of the CONSORT guidelines for RCTs [16,21]. The risk of reporting biases of the controlled cohort studies was assessed using the Newcastle Ottawa Scale, yielding an overall score of seven for the study of Bauss et al. [23,28]. The risk of reporting biases assessments for the clinical trials, conducted using the ROBINS-1 scale, showed a moderate overall risk of bias for Faramarzi et al. and a moderate to serious overall risk of bias for Oesterle and colleagues [15,22,27]. The overall risk of bias of the nine case series was evaluated using the proposed scale modifications tool with moderate risk of bias and a low level of evidence [22,26]. The results of the ROB assessments are presented in Table 3.

## 4. Discussion

This is the first systematic review on the effectiveness of orthodontic forced eruption (OFE) conducted according to well-established guidelines. Although the findings of the present systematic review suggest that OFE is a feasible method to salvage initially non-restorable teeth with CRFs, the majority of studies included here were case series with a low level of evidence. Further, OFE was used to improve esthetics successfully due to periodontal tissue migration or, if coronal migration was not intended, it could be prevented by fiberotomy, as shown in the one identified RCT [16]. Various OFE methods are available for the extrusion of teeth with subgingival defects, depending on the initial clinical conditions and the goal of treatment. The three main methods are outlined below.

### 4.1. OFE without Migration of the Gingival Margin and Alveolar Bone

OFE without migration of the gingiva and alveolar bone is one way to pretreat crown-root fractured teeth before restoration. In these clinical cases, the gingival line should be preserved, there must be sufficient bone around the remaining root, and the remaining root must be extruded far enough to allow for prosthetic restoration with an adequate ferrule design. The aim of fiberotomy is to section the supracrestal gingival fibers. It can prevent the coronal migration of periodontal tissues after OFE and must be performed at the beginning of OFE and repeated weekly or biweekly [15,16]. The use of strong extrusion forces (>0.6 N) is another way to accomplish OFE without hard and soft tissue migration. In the most representative case series (N = 32 patients), OFE was performed using a strong extrusive force of 0.6–0.7 N, which was applied via sectional orthodontic appliances (0.16 × 0.16 inch Elgiloy wires) [29]. The authors reported an average extrusion distance of around 1.0 to 1.5 mm per week. In most cases, a simple gingivectomy was deemed necessary after OFE. However, a slight relapse occurred in three cases, and severe root resorption was noted in one case [29]. Interestingly, the authors of all other case reports with an average number of three patients per case series of OFE with forces less than 0.6 N observed a similar extrusion distance of 1.5 mm/week and the need for post-extrusive periodontal surgery with full-thickness flap elevation. Moreover, the fiberotomy has no influence on the speed of the tooth extrusion. The term “rapid forced extrusion” published by Malmgren et al. and Pontoriero et al. has a purely historical meaning and is related to resection of supracrestal fibers without any influence on the speed of extrusion [29,32,33].

OFEs with moderate forces <0.6 N were usually performed with round nickel–titanium wires [7]. Furthermore, the RCT findings indicate that treatment time is reduced in cases of OFE immediately after open-flap surgery compared to OFE followed by periodontal surgery [34]. Although performing OFE with strong extrusive forces might achieve faster extrusion, this approach results in a need for subsequent gingivectomy [29]. Additionally, OFE for re-alignment of the level of the marginal gingiva obviates the need for even more invasive surgical interventions. More RCTs with large sample sizes are needed to confirm the findings of this systematic review.

### 4.2. OFE with Gingival Migration and Slight Alveolar Bone Migration

Another purpose of OFE is to induce tooth extrusion with gingival migration and slight alveolar bone migration. This approach is used to improve the periodontal status of traumatized teeth presenting with localized bone loss. Indications include teeth with vertical and horizontal alveolar defects characterized by gingival recession and/or papilla loss, which usually occur in association with periodontal disease or dental trauma. With this OFE protocol, soft tissue migration and minimal hard tissue migration are stimulated by applying weak (0.2–0.3 N) to moderate (>0.3 N and <0.6 N) extrusive force via round nickel–titanium wires. Clinically, this conventional method of extrusion can be performed with a partial appliance in one arch.

The nine case series included in this analysis reported a mean extrusion distance of 1.5 mm per week. This is in accordance with the findings of the one prospective clinical trial and other authors, who reported rates of approximately 1 to 2 mm in two weeks [15,35,36]. Interestingly, among the case series, there was no difference in extrusion distance between OFE using wires versus OFE using posts in combination with elastics, as well as posts and wires (Figure 2B). However, there was a slightly higher tendency for a greater extrusion distance (mm) per week with fixed orthodontic appliances compared with elastics within case series (Figure 2B). The advantage of an extrusion by brackets and wires is the continuous force transmission of the arches to the tooth, as well as an extrusion in orthoaxial direction. An investigation concerning force delivery of NiTi orthodontic arch wire showed that the bond between bracket and wires caused a change of the martensitic plateau into a slope [37]. Interestingly, the wire recovered from greater magnitude of deflection released lower force than one recovered from smaller deflection [37]. In contrast, several studies reported that the force levels of elastics might be corresponding to an elongation of the doubled diameter of the lumen [38]. Furthermore, it has been detected that there is a severe decrease of the applied forces from the elastics (up to 30%) compared to an initial high slope [39,40]. Thus, a prolonged extrusion time might be caused by various levels of extrusion forces during OFE using elastics. Furthermore, analysis of the nine case series showed significant correlations due to shorter extrusion times (up to six weeks) in younger patients (11 to 24 years) versus longer extrusion times per week in adults (up to 10 weeks) (*p* < 0.05). This might be attributable to more dynamic bone activity and metabolism in young patients still in the phases of growth compared to adolescents [41].

### 4.3. OFE with Migration of the Gingival Margin and Alveolar Bone

Orthodontic implant site development (OISD) is a specific indication for this OFE technique. In OISD, bone gain is achieved by slow extrusion of the fractured tooth, and the fractured tooth is only temporarily retained during the extrusion phase [13]. OISD may be of particular benefit to patients with periodontal defects as well as horizontal or vertical bone loss in the anterior region. This forced extrusion technique is used to develop more favorable soft-tissue conditions prior to implant placement and leads to the reduction of pathologic periodontal probing depths [42,43]. The procedure allows the surgeon to place an implant immediately after extraction of the extruded tooth. It was estimated that OISD has an efficacy of more than 70% for bone regeneration and 60% for gingival augmentation [44].

### 4.4. Extrusion and Anchorage Techniques

Around 87% of patients in the included case series were treated with a post (temporary or regular) to achieve more reliable extrusion; 20% of these patients were treated with a post and hook, along with orthodontic appliances with a flexible wire, and 80% with a post and elastics to a sectional fixed wire directly bonded to the adjacent teeth [7,12,31,45]. Sectional orthodontic appliances were used in all case reports [14,36,45]. During extrusion, the bracket should be positioned on the tooth crown as close to the cementoenamel junction as possible [31,36,45]. Another possibility is to bond a wire directly onto the surface of the involved tooth including up to four adjacent teeth to minimize adverse effects [15,27]. Furthermore, the cutting incisal edge of the affected tooth can be shortened up to 1 mm in order to facilitate adequate tooth eruption and to avoid occlusal trauma [7,45].

The use of attractive magnets as an alternative to conventional orthodontic appliances for OFE has been proposed [46]. Depending on the type of magnets used, the investigators found that magnets usually achieved an extrusive force of 0.13 N at a distance of 1 mm, which increased to 0.3 N at a distance of 0.5 mm, and to 0.65 N when the magnets were touching [17]. However, there is no evidence suggesting that magnets improve the outcome of OFE compared to conventional orthodontic appliances. Moreover, well-designed studies on this topic are lacking.

### 4.5. Pulp Vitality and Root Resorption

It was reported that traumatized maxillary incisors with severe periodontal tissue injuries have a higher risk of pulp necrosis with OFE than those without orthodontic treatment [28]. A histological study revealed that the pulpal reaction after OFE could depend on the diameter of the apical foramen in coronal direction [47]. Bauss and colleagues observed no difference in the occurrence of pulp necrosis between 32 teeth with hard tissue injuries treated with OFE using weak extrusion forces (0.2 N) and 68 teeth with hard tissue injuries and without OFE treatment [28]. Moreover, pulp vitality tests were recommended at least until the end of retention period, especially for teeth with severe periodontal tissue injuries [28].

In most of the case series, the operators diagnosed an unfavorable pulp prognosis and performed root canal treatment [29]. In fact, endodontic treatment might have been initiated in order to enable root canal anchorage for complication-free extrusion. According to current guidelines, (partial) pulpotomy is considered the treatment of choice for fractured teeth with pulp exposure [48,49]. This approach is only feasible when there is sufficient dental hard tissue to bond a bracket and no need for an intracanal anchorage. Vital pulp treatment is likely to promote hard tissue formation over the healed pulp tissue. While full pulpotomy may lead to further pulp canal obliteration, there does not seem to be an increased risk following partial pulpotomy [50]. This systematic review does not provide evidence regarding whether OFE increases the risk of pulp necrosis in teeth with pulp exposure and subsequent vital pulp treatment.

Root resorption does not seem to be a main issue associated with OFE. A case of severe resorption was diagnosed by Malmgren et al. one year after OFE, but the resorption did not progress during the following two years of observation. Moreover, there was no correlation between the degree of extrusion and the occurrence of root resorption [29]. No further cases of severe root resorption were reported in the other studies included in this review. Additional studies are needed to further investigate the clinical relevance of pulp vitality to OFE and the possible association of root resorption with OFE.

### 4.6. Stabilization after OFE

An adequate period of retention to stabilize the root in its new position following OFE is mandatory in all cases. A rectangular stainless steel wire was used to prevent relapse in most cases reported in the evaluated case series [36,45,51]. This systematic review could not generate reliable data on the ideal stabilization period after OFE, but a minimum retention duration of 8 weeks was adopted in most cases, which is in agreement with common recommendations [16].

## 5. Conclusions

According to the evidence revealed in the studies included in this review, OFE seems to be a predictable and feasible pre-treatment in traumatized teeth with subgingival fracture obtaining both their preservation and favorable esthetics. However, within the limitations of the present review, the evidence retrieved from the included nine case series and four studies indicates a successful OFE with a mean rate of 1.5 mm per week. Three key OFE protocols for traumatized teeth are available, depending on whether the aim of treatment is to induce or prevent coronal migration of marginal gingiva and/or alveolar bone. The use of stronger extrusion forces might enable faster extrusion, but usually requires subsequent gingivectomy. Additionally, OFE for re-alignment of the level of the marginal gingiva obviates the need for even more invasive surgical interventions. Furthermore, OFE is a possible treatment option to preserve subgingival fractured permanent anterior teeth of all age groups. Finally, the evidence for both the beneficial and the adverse effects (e.g., root resorption, pulp necrosis) of OFE is rather low. More RCTs with larger sample sizes are needed to confirm the findings of this systematic review.

## Figures and Tables

**Table 1:** Study design according to PICO.

Criteria	Specification
Research question (focus)	“Is orthodontic forced eruption a successful and effective pre-restorative therapy of non-restorable permanent teeth with subgingival dental hard tissue defects after trauma?”
Population	Patients with non-restorable permanent teeth with subgingival defects due to trauma or caries
Intervention	Orthodontic forced eruption techniques (including anchorage and stabilization) of the affected tooth
Comparator	Other treatment options
Outcomes	Pulp vitality, root resorption, soft and hard tissue changes, amount of tooth movement of the affected tooth

**Table 2:** List of search terms and their combinations used in the search strategy.

Database	Search Terms
PubMed	((((((crown tooth[MeSH Terms]) OR tooth root[MeSH Terms]) OR fracture*) OR crown*) OR root*)) AND ((tooth extrusion[MeSH Terms]) OR tooth eruption[MeSH Terms] OR orthodontic forced eruption[MeSH Terms] OR orthodontic extrusion[MeSH Terms])
Cochrane Library	crown tooth OR root tooth OR fracture/fractures OR crown/crowns OR root/roots AND tooth extrusion OR tooth eruption OR orthodontic forced eruption OR orthodontic extrusion
Embase	crown tooth OR root tooth OR fracture/fractures OR crown/crowns OR root/roots AND orthodontic extrusion OR tooth eruption OR orthodontic forced eruption

**Table 3:** Risk of reporting bias assessment for case series by Murad et al. (2018) [26].

Assessing Risk of Reporting Biases for Case Series by Murad et al. (2018)	Bielak et al. (1982)	Biggerstaff et al. (1986)	Heithersay et al. (1973)	Ingber et al. (1976)	Ivey et al. (1980)	Levine et al. (1997)	Malmgren et al. (1991)	Pontoriero et al. (1987)	Simon et al. (1984)
Selection bias—selection method	−	−	−	−	−	−	−	−	−
Ascertainment—exposure	+	+	+	+	+	+	+	−	−
Ascertainment—outcome	+	+	+	+	+	+	+	+	+
Causality—alternatives	−	−	−	−	−	−	−	−	−
Causality—challenge	+	+	+	+	+	+	+	+	+
Causality—effect	+	+	+	+	+	+	+	+	+
Causality—follow-up	−	−	−	−	−	+	+	−	−
Reporting bias—details	+	+	+	+	+	+	+	+	+
Level of evidence	4	4	4	4	4	4	4	4	4
Overall risk of bias	5	5	5	5	5	6	6	4	4

**Table 4:** Characteristics of the selected studies and their major findings. Abbreviations: FTF, full-thickness flap; OFE, orthodontic forced eruption.

Study	Study Design	Number of Treated Teeth and Defect Type	Orthodontic Appliance	Extrusion Force	Amount of Tooth Movement (mm/Week)	Fiberotomy	Periodontal Surgery	Soft and Hard Tissue Changes after OFE
Bauss et al. (2010)	Retrospective cohort study	Group 1: 32 (trauma) Orthodontic therapy + OFE after trauma Group 2: Orthodontic therapy without trauma Group 3: 68 (trauma) Trauma without orthodontic therapy/OFE	Sectional: Utility arch	Weak (0.2–0.3 N)	Not reported	No	No	Not reported
Bielak et al. (1982)	Case series	3 (trauma)	Sectional: bracket position more apical	Moderate (<0.6 N)	1.9	No	FTF after OFE	Marginal gingiva and alveolar bone more coronal
Biggerstaff et al. (1986)	Case series	3 (trauma, caries)	Sectional: bracket position more apical	Moderate (<0.6 N)	1.6	No	FTF after OFE	Marginal gingiva and alveolar bone more coronal
Carvalho et al. (2006)	RCT	20 (trauma) fractured maxillary incisors Group A: OFE + fiberotomy and root planing Group B: OFE	Sectional: bracket position more apical	Moderate (<0.5 N)	Not reported	Group A: yes (weekly until retention time) Group B: no	FTF after OFE in group B	Marginal gingiva: unaltered in group A Marginal gingiva and alveolar bone more coronal in group B
Faramarzi et al. (2017)	Prospective clinical trial, 1 case report	20 (caries, trauma)	Sectional: post included a hook and elastics; fixed sectional horizontal wire	Moderate (<0.6 N)	Not reported	Nd:YAG laser fiberotomy: 48 h after start of OFE; performed every 2 weeks	No	No migration of gingiva or alveolar bone
Heithersay et al. (1973)	Case series	3 (trauma)	Sectional: temporary post and flexible spring; twistflex wire	Moderate (<0.6 N)	1.5	No	FTF after OFE	Marginal gingiva and alveolar bone more coronal
Ingber et al. (1976)	Case series	4 (trauma)	Sectional: bracket position more apical	Moderate (<0.6 N)	1.7	No	FTF and slight osteotomy after OFE	Marginal gingiva and alveolar bone more coronal
Ivey et al. (1980)	Case series	4 (trauma, caries)	Sectional: bracket position more apical	Moderate (<0.6 N)	1.3	No	FTF after OFE	Marginal gingiva and alveolar bone more coronal
Levine et al. (1997)	Case series	4 (trauma, caries)	Sectional: bracket position more apical (3 cases); post and elastic (1 case)	Moderate (<0.6 N)	1.8	No	FTF after OFE	Marginal gingiva and alveolar bone more coronal
Malmgren et al. (1991)	Case series	32 (trauma)	Sectional: 0.16 × 0.16 inch Elgiloy	Strong (>0.6 N)	1.3	No	Gingivectomy after OFE	No migration of gingiva or alveolar bone
Oesterle et al. (1991)	Retrospective clinical trial	>100 (trauma, caries)	(a) temporary crown with a self-threading pin (b) hook bent from 0.020-inch stainless steel wire (c) elastic chain or tie	Weak (0.2–0.3 N)	Not reported	No	FTF after OFE	Marginal gingiva and alveolar bone more coronal
Pontoriero et al. (1987)	Case series	3 (caries)	Sectional: post with hook and elastic; fixed wire	Moderate (<0.6 N)	1.3	Case 1: no Case 2: mesially, distally without fiberotomy (control) Case 3: circular; weekly during extrusion	Case 1: FTF and osteotomy Case 2: FTF and osteotomy distally of 14 Case 3: Gingivectomy	1. Case: Marginal gingiva and alveolar bone more coronal 2. Case: More coronal (raised and beveled) distally instead of mesially Case 3: No migration of gingiva or alveolar bone
Simon et al. (1984)	Case series	3 (trauma, caries)	Sectional: post with hook and elastic, fixed sectional horizontal wire	Moderate (<0.6 N)	Not reported	No	No	Migration of marginal gingiva, alveolar bone more coronal
